# Genetic alteration profiling of patients with resected squamous cell lung carcinomas

**DOI:** 10.18632/oncotarget.9096

**Published:** 2016-04-29

**Authors:** Dan Tao, Xiaohong Han, Ningning Zhang, Dongmei Lin, Di Wu, Xinxin Zhu, Wenya Song, Yuankai Shi

**Affiliations:** ^1^ Department of Medical Oncology, National Cancer Center/Cancer Hospital, Beijing Key Laboratory of Clinical Study on Anticancer Molecular Targeted Drugs, Chinese Academy of Medical Sciences & Peking Union Medical College, Beijing, China; ^2^ Department of Pathology, National Cancer Center/Cancer Hospital, Chinese Academy of Medical Sciences & Peking Union Medical College, Beijing, China

**Keywords:** squamous cell lung carcinoma, genetic alteration, next-generation sequencing, fluorescence in situ hybridization, targeted therapy

## Abstract

In this study, we analyzed the genetic profiles of squamous cell lung carcinoma (SqCLC) to identify potential therapeutic targets. Approximately 2,800 COSMIC mutations from 50 genes were determined by next-generation sequencing. Amplification/deletion of *SOX2*, *CDKN2A*, *PTEN*, *FGFR1*, *EGFR*, *CCND1*, *HER2* and *PDGFRA* were detected by FISH and expression of VEGFR2, PD-L1 and PTEN were examined by IHC. One hundred and fifty-seven samples of SqCLC were collected. Somatic mutations was identified in 73.9% of cases, with *TP53* (56.1%), *CDKN2A* (8.9%), *PIK3CA* (8.9%), *KRAS* (4.5%) and *EGFR* (3.2%). Gene copy number alterations were identified in 75.8% of cases, including *SOX2* amplification (31.2%), *CDKN2A* deletion (21.7%), *PTEN* deletion (16.6%), *FGFR1* amplification (15.9%), *EGFR* amplification (14.0%), *CCND1* amplification (14.0%), *HER2* amplification (9.6%) and *PDGFRA* amplification (7.6%). Positive expression of VEGFR2 and PD-L1 and loss of PTEN expression were observed in 80.5%, 47.2%, and 42.7% of cases, respectively. Multivariate analysis showed that positive expression of PD-L1 was an independent favorable prognostic factor for DFS (HR = 0.610; *P* = 0.044). In conclusion, nearly all (93.6%) SqCLC cases harbored at least one potential druggable target. The findings of this study could facilitate the identification of therapeutic target candidates for precision medicine of SqCLC.

## INTRODUCTION

Lung cancer is the leading cause of cancer-related death worldwide [1]. Non-small cell lung cancer (NSCLC) accounts for nearly 85% of newly diagnosed lung cancers, and among these, 30% are squamous cell lung carcinoma (SqCLC) [2, 3]. The identification of therapeutically tractable molecular targets, particularly epidermal growth factor receptor (EGFR) mutation and anaplastic lymphoma kinase (ALK) rearrangement, has led to the dramatic improvement in personalized therapy for lung adenocarcinoma [4–6]. However, in contrast to lung adenocarcinoma, still now no approved targeted therapies are available for SqCLC. The targeted drugs developed for lung adenocarcinoma such as *EGFR* tyrosine kinase inhibitor (EGFR-TKI) and ALK inhibitor, are largely ineffective against SqCLC [7]. Therefore, identifying novel molecular targets for the personalized therapies of SqCLC became a top research priority.

Previous studies have identified several genetic alterations related to SqCLC, such as mutations in *TP53*, *EGFRvIII*, *PIK3CA*, *NRF2*, *PTEN* and *DDR2*, as well as amplification of *FGFR1*, *PDGFRA*, *SOX2*, *p63* and *PIK3CA*, which were considered as candidate driver genes for targeted therapy [2, 8–13]. However, these genes have yet to translate into clinically useful prognostic or predictive biomarkers in SqCLC. Although recent next-generation sequencing (NGS) studies have demonstrated a comprehensive genomic characterization of SqCLCs in both Caucasian and Korean patients [7, 14], the data of these studies can't explain all SqCLC patients. In addition, the genomic characterization of SqCLCs in Chinese patients is still not clear. Therefore, additional evidence from genomic level studies of SqCLC is needed to facilitate the identification of potential therapeutic targets in SqCLC.

In this study, we comprehensively analyzed mutations in a large cohort of patients with resected squamous cell lung carcinoma by using NGS techniques. Copy number alteration of *FGFR1*, *EGFR*, *HER2*, *PDGFRA*, *CCND1*, *SOX2*, *CDKN2A* and *PTEN* were further examined by fluorescence in situ hybridization (FISH). In addition, expression status of PTEN, PD-L1 and VEGFR2 were analyzed by immunohistochemistry (IHC). The aim of this study was to reveal the genomic characterization of SqCLC and to identify potentially clinical actionable molecular targets for this subset of patients.

## RESULTS

### Patient characteristics

A total of 157 patients with surgically resected SqCLC were analyzed. The clinicopathologic characteristics of the patients are summarized in Table 1. Patients had a median age of 59 years old (range 42 - 83 years). The majority of patients were male (92.4%), and former (15.9%) or current (73.2%) smokers. All samples were primary resection specimens, with evidence of pathologic stage I in 33.8%, stage II in 29.3%, stage III in 36.3%, and stage IV in 0.6%. The survival data was available in all 157 patients with SqCLC. The median follow-up time was 41.4 months.

**Table 1:** Clinicopathological characteristics of 157 patients

Variables	No.	%
Sex		
Male	145	92.4
Female	12	7.6
Age (years)		
< 65	107	68.2
≥ 65	50	31.8
Smoking stataus		
Never smoker	17	10.8
Former smoker	25	15.9
Current smoker	115	73.2
Histology		
Squamous	151	96.2
Adenosquamous	1	0.6
Squamous with small cell	2	1.3
Squamous with basaloid	3	1.9
Differentiation		
Well	9	5.7
Moderate	73	46.5
Poor	75	47.8
Stage		
I	53	33.8
II	46	29.3
III	57	36.3
IV	1	0.6

### Analysis of somatic gene mutations

An adequate library was obtained from all 157 samples for Ion Torrent sequencing. The mean read length was 105 bp, constituting an average of approximately 32 Mb of sequence per sample. With normalization to 330,000 reads per specimen, there was an average of 1602 reads per amplicon (range, 131 to 6883), and 206/207 (99.5%) amplicons averaged at least 100 reads (Supplementary Figure S1A), and 201/207 (97.1%) amplicons averaged at least 300 reads (Supplementary Figure S1B).

**Figure 1:**
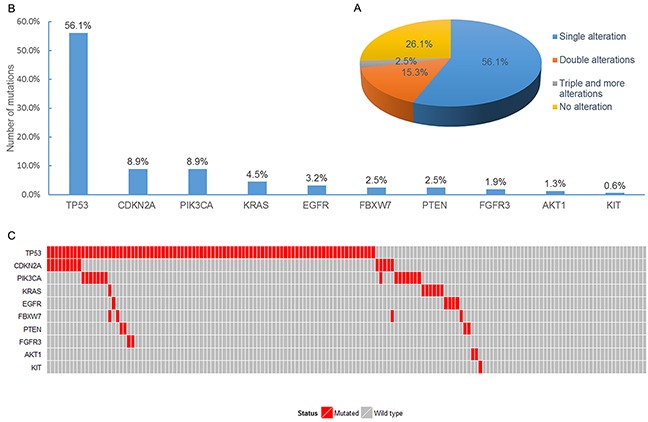
Analysis of somatic mutations in FFPE specimens from SqCLC patients **A.** The pie chart demonstrated the distribution for the number of mutations detected in specimens. **B.** Number of mutations in each of the 10 mutated genes for the 157 specimens. **C.** Mutational profiles for all the patients analyzed in this study.

Totally, somatic mutations were found in 116 tumors (73.9%). Among the 116 tumors with mutation, 88 tumors (56.1%) harbored a single mutation in a single gene, 24 tumors (15.3%) had two mutations (either in the same gene or two different genes), three tumors (1.9%) showed three mutations, and one patient (0.6%) harbored five mutations (Figure 1A).

Of the 50 genes analyzed, ten genes showed mutations (unique or associated with other mutations): *TP53* (88 cases, 56.1%), *CDKN2A* (14 cases, 8.9%), *PIK3CA* (14 cases, 8.9%), *KRAS* (7 cases, 4.5%), *EGFR* (5 cases, 3.2%), *FBXW7* (4 cases, 2.5%), *PTEN* (4 cases, 2.5%), *FGFR3* (3 cases, 1.9%), *AKT1* (2 cases, 1.3%), and *KIT* (1 case, 0.6%) (Figure 1B). Mutation profiles for patients harboring at least one mutation are illustrated in Figure 1C. All *EGFR* and *KRAS* mutations were mutually exclusive. *TP53* was mutated in 22 of 24 cases (91.6%) with co-mutations, making it the most common mutated gene in cases of co-mutations. One patient with *EGFR* mutation also harbored *TP53* mutation. In addition, one case harbored five mutations involving three genes (including double mutations of *TP53* and *KRAS*, and single mutation of *FBXW7*). Details of the identified mutations are summarized in Supplementary Table S1.

The relationships between mutational status of SqCLC and clinicopathological characteristics were analyzed (Supplementary Table S2). The frequency of *EGFR* mutation was significantly higher in female than in male (33.3% *vs.* 0.7%, *P* < 0.001), and was significant higher in never smokers than in smokers (23.5% *vs.* 0.7%, *P* < 0.001). Conversely, *TP53* mutations were significantly more common in men than in women (58.6% *vs.* 25.0%, *P* = 0.024), and more common in smokers than in non-smokers (59.3% *vs.* 29.4%, *P* = 0.019). No significant association was found between other gene mutations and clinicopathologic variables in this cohort of patients with SqCLC. No significant prognostic impact of these gene mutations was found in the univariate and multivariate survival analysis (Supplementary Table S3).

### Characterization of copy number alterations

**Figure 2:**
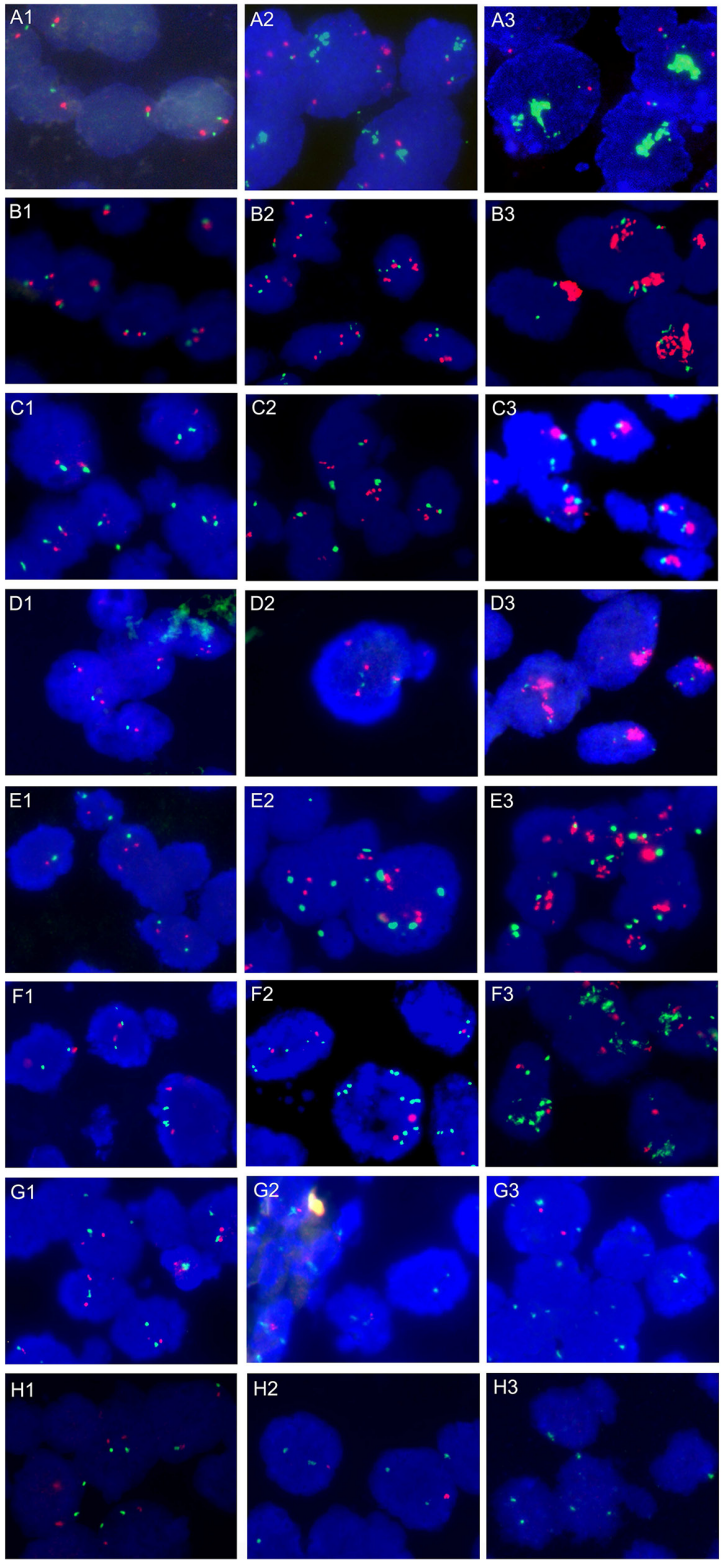
Representative photographs of FISH assay results **A.** FGFR1/CEP8 signal patterns: A1, Disomy; A2, Low-level *FGFR1* amplification; A3, High-level *FGFR1* amplification with large clusters. **B.** EGFR/CEP7 signal patterns: B1, Disomy; B2, Low-level *EGFR* amplification; B3, High-level *EGFR* amplification with large clusters. **C.** HER2/CEP17 signal patterns: C1, Disomy; C2, Low-level *HER2* amplification; C3, High-level *HER2* amplification with large clusters. **D.** PDGFRA/CEP4 signal patterns: D1, Disomy; D2, Low-level *PDGFRA* amplification; D3, High-level *PDGFRA* amplification with large clusters. **E.** CCND1/CEP11 signal patterns: E1, Disomy; E2, Low-level *CCND1* amplification; E3, High-level *CCND1* amplification with large clusters. **F.** SOX2/CEP3 signal patterns: F1, Disomy; F2, Low-level *SOX2* amplification; F3, High-level *SOX2* amplification with large clusters. **G.** CDKN2A/CEP9 signal patterns: G1, Disomy; G2, Homozygous deletion of *CDKN2A* with internal positive control; G3, Heterogeneous intratumoral *CDKN2A* gene deletion. **H.** PTEN/CEP10 signal patterns: H1, Disomy; H2, Hemizygous deletion of *PTEN* gene; H3, Homozygous deletion of *PTEN* gene.

**Figure 3:**
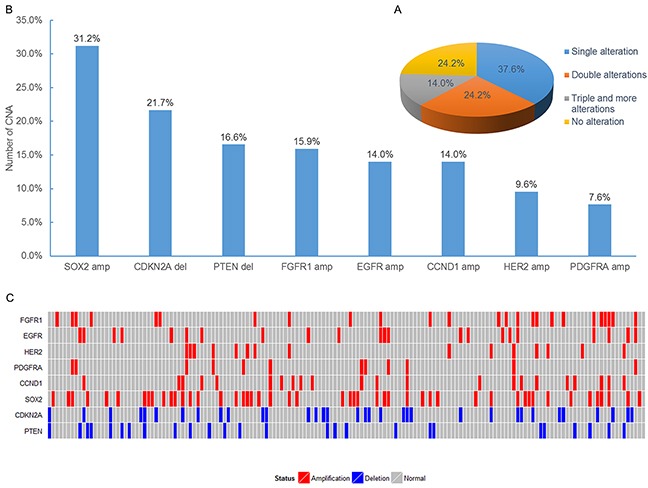
Analysis of copy number alterations in FFPE specimens from SqCLC patients **A.** The pie chart demonstrated the distribution for the number of copy number alterations detected in specimens. **B.** Number of copy number alterations in each of the 8 analyzed genes for the 157 specimens. **C.** Profiles of copy number alteration for all the patients analyzed in this study.

Gene copy number alterations (CNA) of *FGFR1, EGFR, HER2, PDGFRA, CCND1, SOX2, CDKN2A,* and *PTEN* were evaluated by FISH. All 157 specimens were tested successfully. Representative images of FISH results are illustrated in Figure 2. One hundred and nineteen patients (75.8%) were identified with at least one gene copy number alteration. Of the 157 patients, 59 (37.6%) harbored single copy number alterations, 38 (24.2%) harbored double copy number alterations, and 22 (14.0%) showed triple and more copy number alterations (Figure 3A). Overall, we identified *FGFR1* amplification in 25 patients (15.9%), *EGFR* amplification in 22 patients (14.0%), *HER2* amplification in 15 patients (9.6%), *PDGFRA* amplification in 12 patients (7.6%), *CCND1* amplification in 22 patients (14.0%), *SOX2* amplification in 49 patients (31.2%), *CDKN2A* deletion in 34 patients (21.7%), and *PTEN* deletion in 26 patients (16.6%) (Figure 3B). CNA profile of these genes was demonstrated in Figure 3C. The frequency of *CDKN2A* deletion in squamous cell carcinoma combined with other components was significantly higher than that in pure squamous cell carcinoma (66.7% vs. 19.9%, *P* = 0.026) (Supplementary Table S4). No significant relationships between CNA of the eight genes and DFS and OS were observed in univariate and multivariate survival analysis (Supplementary Table S3).

### Expression of PTEN, PD-L1 and VEGFR2

**Figure 4:**
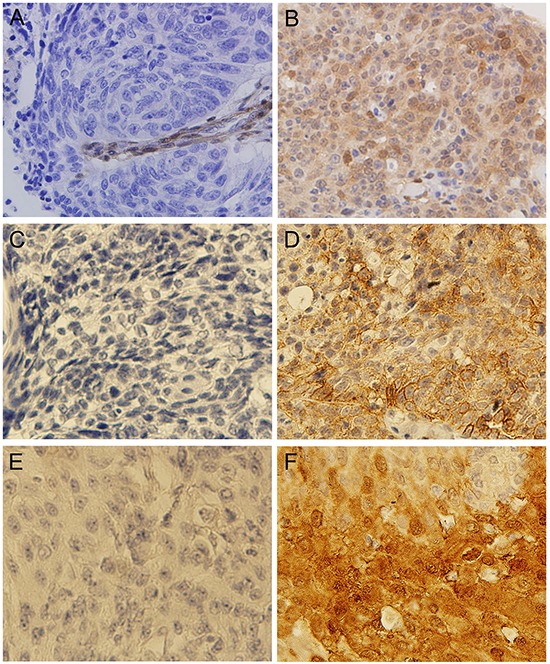
Representative photographs of IHC assay results **A.** Tumor showing lack of staining for PTEN; **B.** Tumor showing positive staining for PTEN; **C.** Tumor showing lack of staining for PD-L1; **D.** Tumor showing positive staining for PD-L1; **E.** Tumor showing lack of staining for VEGFR2; **F.** Tumor showing positive staining for VEGFR2.

**Figure 5:**
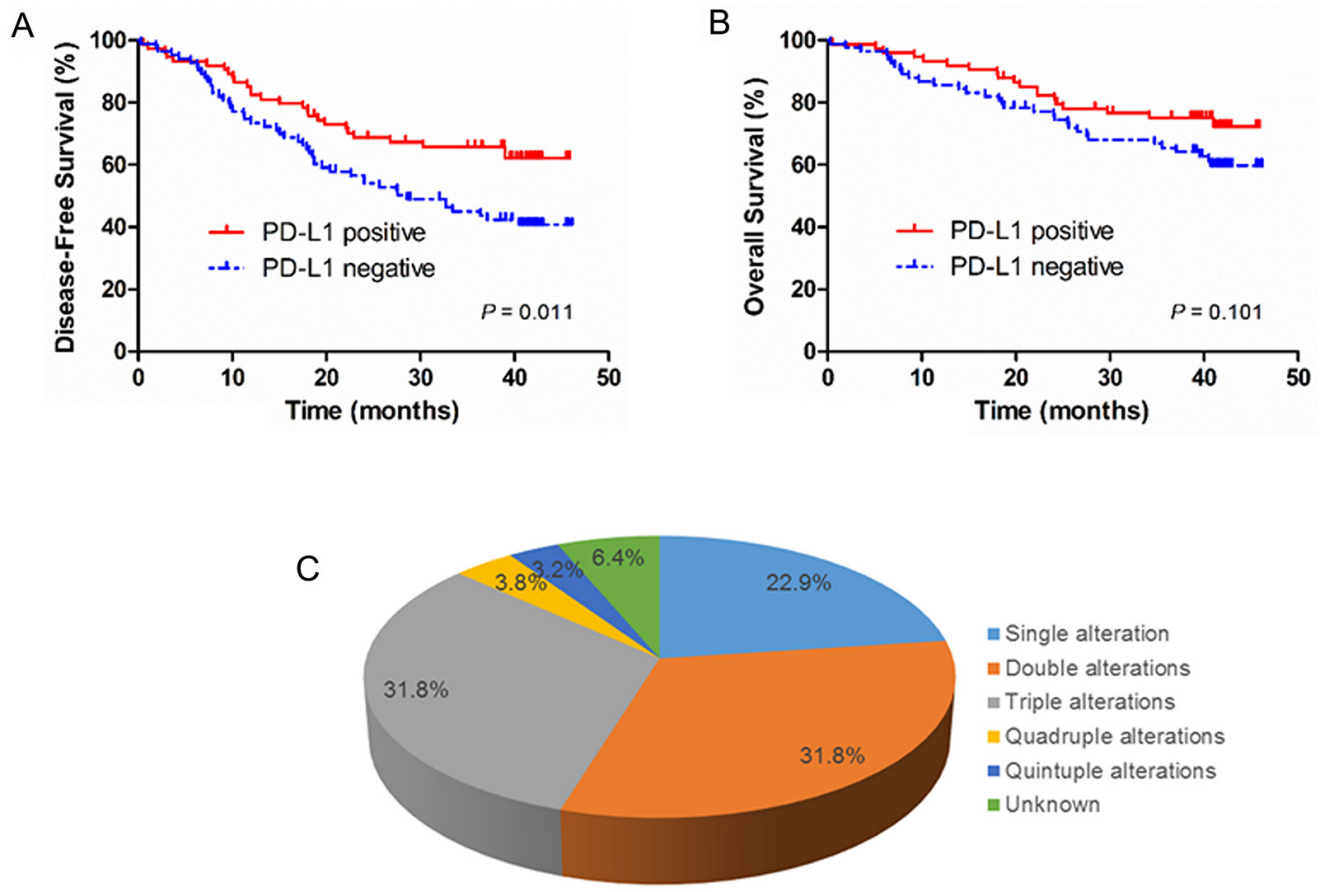
Kaplan–Meier DFS and OS curves based on PD-L1 immunohistochemistry status and the distribution for the potential therapeutic targets detected in SqCLC **A.** Disease free survival (DFS) rates of SqCLC patients by PD-L1 expression status; **B.** Overall survival (OS) rates of SqCLC patients by PD-L1 expression status. **C.** The pie chart demonstrated the distribution for the potential therapeutic targets detected in SqCLC.

In addition to the analysis of mutations and CNAs in these patients, we also evaluated protein expression of PTEN, PD-L1 and VEGFR2 by IHC. Loss of PTEN expression, positive expression of PD-L1 and VEGFR2 were observed in 67 of 157 cases (42.7%), 75 of 157 cases (47.2%), and 128 of 157 cases (80.5%), respectively (Figure 4 and Supplementary Table S5). There was a borderline significance of higher prevalence (*P* = 0.05) of loss of PTEN expression in elderly patients (age≥65 years) (54.0%) than in younger patients (37.4%). In addition, loss of PTEN expression was significantly associated with *PTEN* mutation, *FGFR1* amplification, *HER2* amplification, *CCND1* amplification, and *CDKN2A* deletion (Supplementary Table S6). The incidence of positive expression of PD-L1 was significantly higher in early-stage (I+II) patients than in relatively advanced-stage (III+IV) patients (54.5% *vs.* 34.5%, *P* = 0.015). In univariate survival analysis, positive expression of PD-L1 was associated with a significantly longer DFS (*P* = 0.011) (Figure 5A). Multivariate survival analysis showed that positive expression of PD-L1 was an independent favorable prognostic factor for DFS (HR = 0.610; *P* = 0.044) after adjusting for age, tumor differentiation, and TNM stage (Supplementary Table S3). However, positive expression of PD-L1 was not a significant prognostic factor for OS (Figure 5B). No significant associations of expression status of PTEN and VEGFR2 with DFS and OS were found (Supplementary Table S3).

### Potential therapeutic targets

In our study, we analyzed two common genomic alterations in adenocarcinomas of the lung: *EGFR* and *KRAS* mutations. There were five patients harboring *EGFR* mutations, two individuals demonstrated a canonical exon 19 deletion and one individual demonstrated L858R mutation in exon 21, whereas one patient demonstrated a S768I mutation in exon 19 and one patient demonstrated an exon 20 insertion. Eight individuals harbored a *KRAS* mutation, with 5 G12D, 1 G12C, 1 G12V, and 1 A59E. However, *EGFR* amplifications were found in 14.0% of patients, which confers sensitivity to erlotinib and gefitinib [15].

The presence of novel potential therapeutic targets in SqCLC was suggested by the observation that 100% (157 out of 157) of tumors contain one or more molecular abnormalities, including mutation, amplification/deletion, and positive/negative expression. The potential therapeutic targets were selected based on several features, including availability of targeted therapeutic agents approved by US FDA or one being studied in current clinical trials. Fourteen potential therapeutic targets were selected for analysis, including *EGFR*, *KRAS*, *PIK3CA*, *PTEN*, *AKT1*, *FGFR3*, or *KIT* mutations, *FGFR1*, *EGFR*, *HER2* and *PDGFRA* amplifications, *PTEN* deletion, and PD-L1 and VEGFR2 expression. According to those criteria, we identified 147 cases with molecular abnormalities of a potentially targetable gene (93.6%). There were 70.7% (111/157) of patients harboring two or more molecular abnormalities (Figure 5C).

## DISCUSSION

In recent years, the molecular characterization of lung adenocarcinoma has been well studied. However, the profile of the second common lung cancer, squamous cell lung cancer, is still not fully investigated. In this study, we performed a comprehensive molecular characterization analysis in a large series of resected SqCLC by using NGS, FISH and IHC, aiming to outline a molecular feature in individual tumors. To our knowledge, this is one of the largest retrospective studies comprehensively profiling molecular alterations in Chinese patients with SqCLC.

Our results revealed *TP53* was the most frequently mutated gene in patients with SqCLC, which reached up to 56.1%. Previous published TCGA's study [7] showed that almost all lung SqCLCs displayed somatic mutation of *TP53* (81%), and the cohort in the Kim's study [14] displayed a similarly high rate of mutations of *TP53* (73%), which were similar to our findings. Different detection methods may cause the difference in frequency of *TP53* mutation between our study and previous reports. Based on these results, there does not seem to be significant ethnic differences in the prevalence of *TP53* mutations and *TP53* mutations may be one of the major genomic alterations for the SqCLC patients. As the most frequently mutated gene in SqCLC, *TP53* gene mutation may represent a potential therapeutic target. To date, a number of small-molecule drugs aiming to reactivate mutant *TP53* or activate wild type *TP53* are being developed and some of them are in phase I trials [16, 17].

Currently, the evidence is conflicting regarding prevalence of *EGFR* and *KRAS* mutations in SqCLC [18–22]. Recently, Rekhtman et al. screened 95 biomarker-verified SqCLC and reported that *EGFR/KRAS* mutations do not occur in pure SqCLC, occasional detection of these mutations in samples diagnosed as “SqCLC” is due to the diagnosis of adenosquamous carcinoma (AD-SQC) and adenocarcinoma [18]. However, another similar study by Miyamae et al. revealed that *EGFR* mutations were present in 3.4% of 87 validated SqCLC specimens [20]. In our study, we found that the frequencies of *EGFR* and *KRAS* mutation were 3.2% and 4.5%, respectively, which were comparable with the previous reports of 3.4% [20] and 4% [22], respectively. In addition, the study conducted by TCGA [7] reported two patients with *EGFR* mutation and one patients with *KRAS* mutation from 178 American SqCLC patients, and Kim's study [14] identified one case with *EGFR* mutation and two cases with *KRAS* mutation from 104 Korean SqCLC patients. These results were consistent with our study. It seemed that in patients with SqCLC, the frequencies of *EGFR* and *KRAS* mutation were similar in different ethnicity. In agreement with previously reported study, *EGFR* and *KRAS* mutations were also mutually exclusive in SqCLC [23].

In the present study, we investigated copy number alterations of *FGFR1, EGFR, HER2, PDGFRA, CCND1, SOX2, CDKN2A* and *PTEN* in a cohort of SqCLC FFPE samples by dual colour FISH. To the best of our knowledge, this is the first and largest study concurrently detecting copy number alterations of these genes by using FISH in SqCLC patients. Of note, recently, *FGFR1* amplification was considered as one of the most potential molecular target for the treatment of patients with SqCLC. Previous studies [24, 25] showed that FGFR inhibitors were effective to block tumor proliferation in a subset of NSCLC cell lines with *FGFR1* amplification and led to significant tumor shrinkage. In our study, we found that 15.9% of SqCLC patients harboring *FGFR1* amplification, similar to previous studies where 16.0 % [26] and 20.0 % [27] of SqCLC were amplified, respectively. Although the copy number alterations of some of these genes detected in this study have been examined by NGS or SNP array, the frequency of amplification/deletion of these genes still remains controversial. FISH was considered to be the gold standard method for the assessment of copy number alteration [28]. The frequencies of amplification/deletion of these genes detected in our study would be relatively more accurate. In our study, the gene copy number alterations (75.8%) was a common events in SqCLC, as well as gene mutations (73.9%), which was comparable to that of other studies have also reported [7, 14].

Loss of PTEN expression has been reported to occur in 20% to 93% of SqCLC [29–33]. In the present study, we found that 42.7% of SqCLC demonstrated loss of PTEN protein expression, which was consist with previously reported studies. In addition, we found PD-L1 expression in 47.2% of SqCLC specimens. In a study analyzing specimen from 76 patients, Rizvi et al. [34] reported PD-L1 expression in 33% of SqCLC. These results are comparable to our findings, although Rizvi et al. [34] used a different antibody clone. The slightly higher percentage of positive specimens in our study may be contributed to the difference in experimental technique. The prognostic value of PD-L1 expression is still now controversial. Mu et al. [35] found that high expression of PD-L1 in lung cancer may contribute to poor prognosis, however, Brahmer et al. [36] reported that the expression of PD-L1 was neither prognostic nor predictive of benefit. Böger et al. [37] found that high expression of PD-L1/PD-1 was associated with a significantly better outcome, and PD-L1 was an independent survival prognosticator in gastric cancer. Simlar to Böger et al.'s report, we also found the patients with positive expression of PD-L1 had a significantly longer DFS, and it was an independent favorable prognostic factor for DFS in SqCLC. VEGFR2 is a valid therapeutic target in lung cancer. Overexpression of VEGFR2 is associated with invasion and metastasis in lung cancer. In present study, 80.5% of cases showed positive expression of VEGFR2, which was similar to previous study [38]. The high prevalence of overexpression of VEGFR2 demonstrated that VEGFR2 overexpression was a common event in tumorigenesis of SqCLC.

Our results revealed that 147 of 157 (93.6%) patients with SqCLC harbored *EGFR*, *KRAS*, *PIK3CA*, *PTEN*, *AKT1*, *FGFR2*, or *KIT* mutations, *FGFR1*, *EGFR*, *HER2*, *PDGFRA* and *PTEN* amplifications/deletion, and PD-L1 and VEGFR2 expression, which were potential druggable targets for anticancer therapy. The EGFR-TKIs, such as gefitinib, erlotinib and icotinib, specifically target *EGFR*, and has demonstrated a significant survival benefit for NSCLC patients with activating *EGFR* mutations [4, 6, 39]. Recently, US FDA approved Keytruda (pembrolizumab) for advanced NSCLC, which was the first drug approved in NSCLC for patients whose tumors express PD-L1. Ramucirumab is a human IgG1 monoclonal antibody that targets the extracellular domain of VEGFR-2, which has been approved by US FDA for second-line treatment for patients with advanced NSCLC [40]. In addition, several agents specially targeting molecular alterations of *PIK3CA*, *PTEN*, *AKT1*, *FGFR2*, *KIT*, *FGFR1*, *HER2*, and *PDGFRA* are expected to be effective for the treatment of SqCLC, and currently are being tested in phase I to III clinical trials [2]. Therefore, identification of these druggable targets in SqCLC could lead to rationally chosen specific targeted therapy.

Clinical success with combination targeted therapy depends on the identification of molecular abnormalities for co-administration of a single or combination of target agents against the detected therapeutic targets [41]. In our study, the results indicated that the co-occurrence of mutation (17.8%) or copy number alterations (38.2%) were identified in a significant proportion of tumor samples. These findings can help to guide the targeted therapies for the SqCLC. This study showed that *TP53* mutations was the most frequent mutations co-existed with activating mutations, such as *EGFR*, *KRAS*, and *PIK3CA*, supporting the rationale for developing combinatorial approaches against *TP53* mutations and the co-existed activating mutations in SqCLC. Further studies are warranted to elucidate the mechanisms of concurrent molecular alterations and improve the efficacy of the targeted therapies for SqCLC.

In conclusion, our study has identified potentially targetable molecular alterations in most SqCLC in a large cohort of Chinese patients. The findings of this study could help to provide insights into the profile of genetic alterations in SqCLC and assist oncologist to match patients with available targeted treatments or clinical trials of new targeted agents in the coming era of precision medicine.

## MATERIALS AND METHODS

### Patient

One hundred and fifty-seven patients with histologically confirmed squamous cell lung carcinoma were collected from Cancer Hospital of Chinese Academy of Medical Sciences (Beijing, China) between March 2011 and November 2011. For all patients, medical records were reviewed to obtain clinical and demographic characteristics, including sex, age, smoking history, histology, tumor differentiation, and tumor stage. The histological classification was done according to the 2004 World Health Organization classification of lung tumors. Tumor staging was performed using the 7th edition of the TNM staging system by the International Union Against Cancer (UICC)/AJCC of 2010. This study was approved by the medical ethics committee of the Cancer Hospital of the Chinese Academy of Medical Sciences.

### Sample preparation and next generation sequencing

DNA was extracted from formalin-fixed, paraffin-embedded (FFPE) tumor samples. Unstained 5-μm-thick tissue sections were deparaffinized, and tumor-rich areas (>20%) were manually microdissected by comparison with an H&E-stained slide from the same block. DNA was extracted using the QIAamp DNA Mini Kit (Qiagen) per the manufacturer's instruction. The extracted DNA was quantified using the Qubit 2.0 (Life Technologies).

The Ion AmpliSeq™ Cancer Hotspot Panel v2 (Life Technologies) was used to generate target amplicon libraries and to explore selected regions of the following 50 oncogenes and tumor suppressor genes (in alphabetical order): *ABL1, AKT1, ALK, APC, ATM, BRAF, CDH1, CDKN2A, CSF1R, CTNNB1, EGFR, ERBB2, ERBB4, EZH2, FBXW7, FGFR1, FGFR2, FGFR3, FLT3, GNA11, GNAS, GNAQ, HNF1A, HRAS, JAK2, JAK3, IDH1, IDH2, KDR, KIT, KRAS, MET, MLH1, MPL, NOTCH1, NPM1, NRAS, PDGFRA, PIK3CA, PTEN, PTPN11, RB1, RET, SMAD4, SMARCB1, SMO, SRC, STK11, TP53, VHL*. Details of the target regions and mutations for the gene panel are available at the supplier's website: http://tools.lifetechnologies.com/downloads/cms_106003.csv.

Briefly, 10 ng of DNA were used for multiplex PCR amplification. Emulsion PCR was performed with OneTouch DL systems (Life Technologies). Library concentration and amplicon size was evaluated by the Agilent 2100 Bioanalyzer (Agilent Technologies) and Agilent BioAnalyzer DNA High-Sensitivity LabChip (Agilent Technologies). Sequencing was performed on the Ion PGM Sequencer (Life Technologies) with the Ion PGM 200 Sequencing Kit according to the manufacturer's instructions on Ion 316 Chips.

Data analysis, including alignment to the hg19 human reference genome and variant calling, was performed using the Torrent Suite Software v.3.0 with a plug-in “variant caller” program (Life Technologies). In order to eliminate error base calling, we conducted three filtering steps to generate final variant calling. The first filter was set at an average depth of total coverage of >100, an each variant coverage of >20, a variant frequency of each sample >5 and *P*-value <0.01. The second filter was set at the base calling of < 5 bases homopolymer tracts and > 3 bases from the terminus of amplicons, because there were false positive mutations at the ends of reads we sometimes observed. The third filtering step was employed by visually examining mutations using Integrative Genomics Viewer (IGV) software (http//www.broadinstitute.org/igv) or Samtools software (http://samtools.sourceforge.net), as well as by filtering out possible strand-specific errors, ie. a mutation was only detected in either “+” or “-” strand, but not in both strands of DNA (Supplementary Figure S2).

The presence of mutations in *EGFR*, *KRAS*, and *PIK3CA* genes detected by Ion Torrent next generation sequencing was confirmed by Sanger's sequencing using an ABI 3500XL Genetic Analyzer (Applied Biosystems, Carlsbad, CA, USA), according to the manufacturer's protocol.

### Gene copy number

To determine the gene copy number of *FGFR1*, *HER2*, *EGFR*, *PDGFRA*, *CCND1*, *SOX2*, *CDKN2A*, and *PTEN*, dual-color FISH was performed on paraffin-embedded tissue sections. Therefore, FISH probes for FGFR1/CEN8 (ZytoVision, Germany), EGFR/CEP 7 (Vysis, USA), HER-2/CEP17 (Vysis, USA), PDGFRA/CHR4 (Empire Genomics, USA), CCND1/CEP 11 (Vysis, USA), SOX2/CEN 3 (ZytoVision, Germany), CDKN2A/CEP 9 (Vysis, USA), PTEN/CEP 10 (Vysis, USA) were used in our study. FISH was performed according to the manufacturers' instructions, respectively. FISH analyses were interpreted by two experienced evaluators (D.T. and D.W.) blinded to the clinical data. At least 100 nuclei per patient were evaluated. Detailed definitions of FISH positivity are described in the Supplementary Methods.

### Immunohistochemistry (IHC)

IHC analyses using antibody of PTEN (138G6, CST; at a dilution of 1:100), PD-L1 (E1L3N, CST; at a dilution of 1:100), and VEGFR2 (55B11, CST; at a dilution of 1:300) were performed on paraffin-embedded tissue sections according to the manufacturers' recommended protocols, respectively. Expression levels were scored semi-quantitatively by two evaluators (D.T. and N.N.Z) who had no prior information about the patients. The definitions of loss of PTEN expression, positive expression of PD-L1 and VEGFR2 were according to the previous literatures [29, 34, 42].

### Statistical analysis

The associations between molecular alterations and clinicopathologic variables were examined by Pearson Chi-square tests (for categorical variables) and Kruskal-Wallis tests (for continuous variables), where appropriate. The overall survival (OS) was defined as the time period from the operation date to the date of death or the end date of follow-up. The disease-free survival (DFS) was calculated from the operation date to the time of disease progression or death from any cause. The Kaplan-Meier method was used to estimate OS and DFS and the differences were compared by the log-rank test. Univariate and multivariate analysis was performed by using Cox proportional hazards regression model. A two-sided *P* value of 0.05 was considered statistically significant. All statistical analyses were performed by using Statistical Package for the Social Sciences Version 17.0 Software (SPSS, Inc., Chicago, Illinois).

## SUPPLEMENTARY FIGURES AND TABLES














