# Supplementary material for: Genetic alteration profiling of patients with resected squamous cell lung carcinomas
Source: Oncotarget. 2016 Apr 29;7(24):36590–601. doi: 10.18632/oncotarget.9096 (PMC5095023; doi:10.18632/oncotarget.9096)
Supplement: Supplementary file 1 [file oncotarget-07-36590-s001.pdf]

SUPPLEMENTARY FIGURES AND TABLES

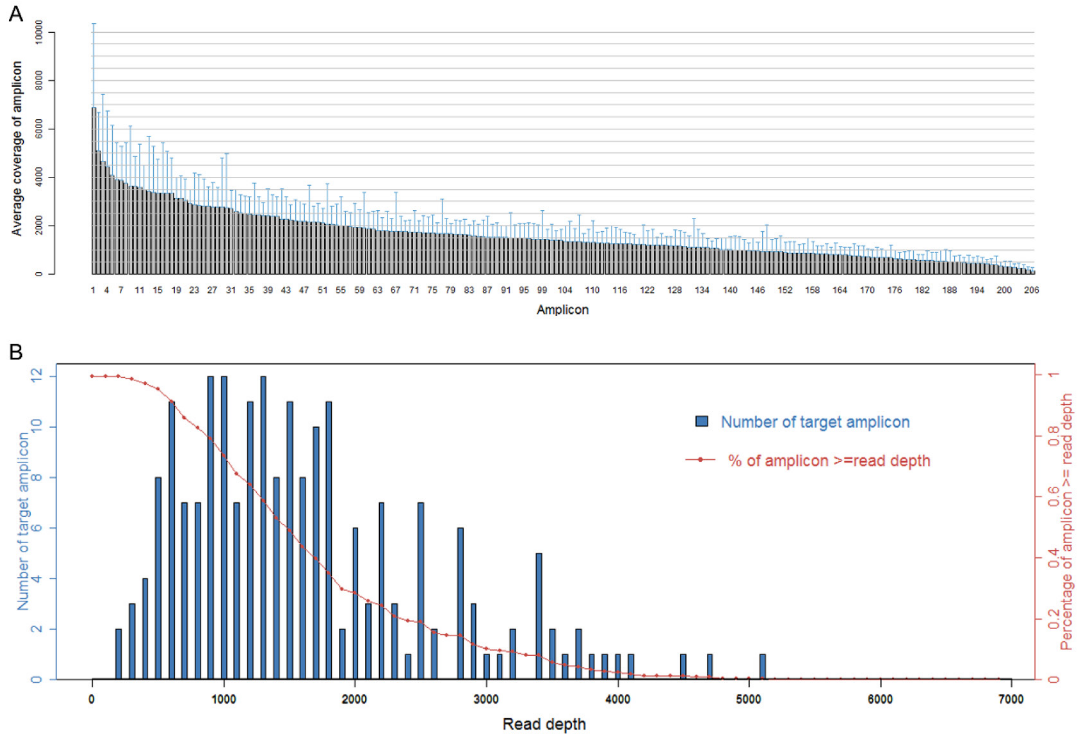

Supplementary Figure S1: Sequence read distribution across 207 amplicons generated from 157 FFPE specimens. A. Distribution of average coverage of each amplicon; data are shown as mean ± SD. **B.** Number of amplicons with a given read depth, sorted in bins of 100 reads. Blue bars represent number of target amplicons within read depth; red line represents percent of target amplicons ≥ read depth.

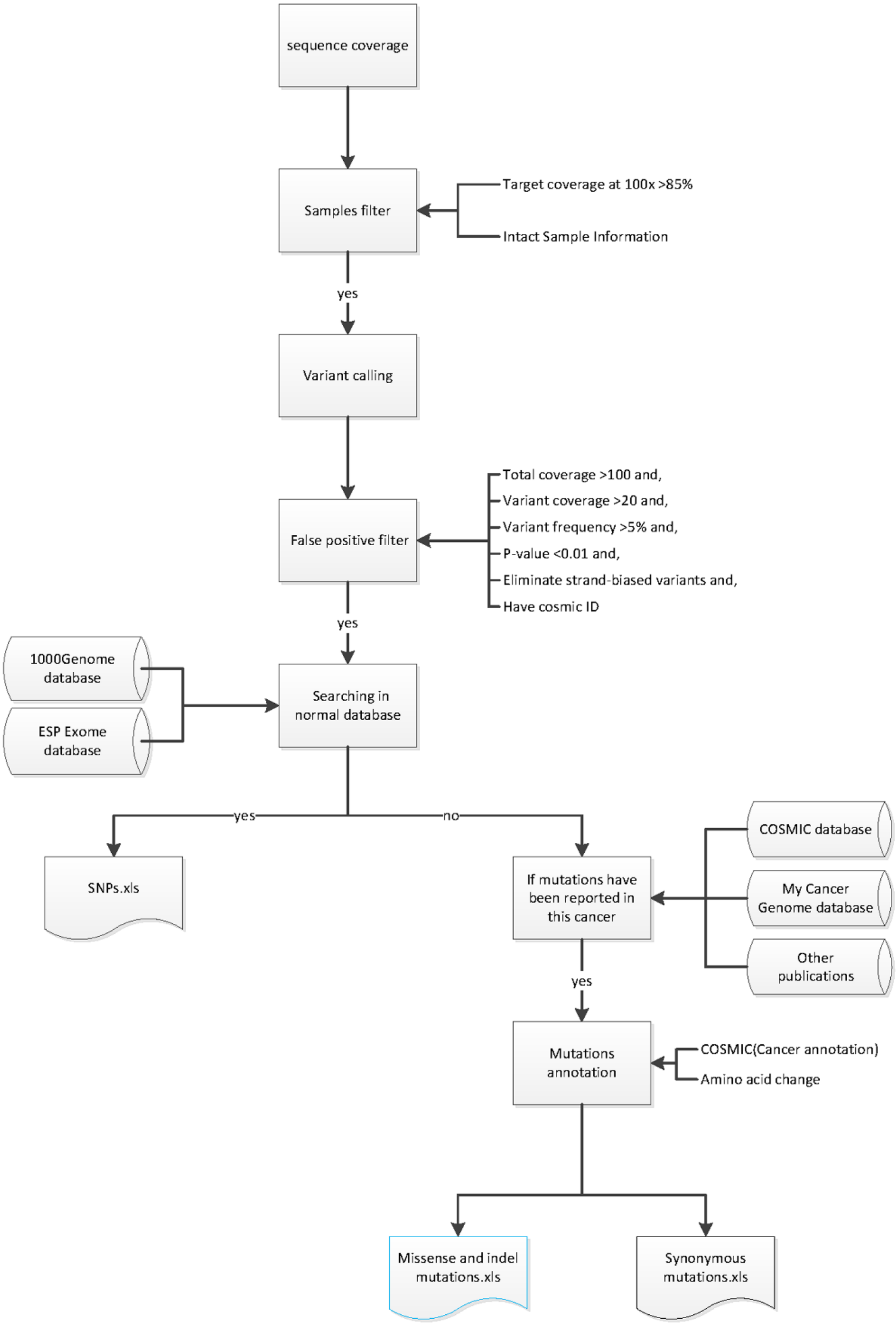

Supplementary Figure S2: Workflow of bioinformatical analysis for Ion Torrent PGM sequencing data.

Supplementary Table S1: Summary of mutations identified by Ion Torrent targeted sequencing in SqCLC.

See Supplementary File 1

Supplementary Table S2: The Association between clinicopathologic characteristics and mutational status of *EGFR*, *KRAS*, *PIK3CA*, *CDKN2A* and *TP53* in 157 SqCLC.

See Supplementary File 2

Supplementary Table S3: Univariate and multivariate analyses of prognostic factors for DFS and OS.

See Supplementary File 3

Supplementary Table S4: The Association between clinicopathologic characteristics and copy number alterations in 157 SqCLC.

See Supplementary File 4

Supplementary Table S5: The Association between clinicopathologic characteristics and expression of PTEN, PD-L1 and VEGFR2 in 157 SqCLC.

See Supplementary File 5

Supplementary Table S6: Correlation between the various main molecular alterations.

See Supplementary File 6

SUPPLEMENTARY METHODS

Definitions of *FGFR1* amplification [1, 2]

Patients were considered as *FGFR1* amplification if satisfied one of the following conditions:

(1) FGFR1/CEN8 ratio ≥2.0;

(2) average number of *FGFR1* signals per tumor cell ≥6;

(3) percentage of tumor cells containing ≥15 *FGFR1* signals or large clusters ≥10%;

(4) percentage of tumor cells containing ≥5 *FGFR1* signals ≥50%.

Cases with condition (1–3) represent a high-level *FGFR1* amplification, and cases with condition (4) represent low-level *FGFR1* amplification.

Definitions of *EGFR* amplification [3]

(1) Low-level *EGFR* amplification defined as percentage of tumor cells containing ≥4 *EGFR* signals ≥40%;

(2) High-level *EGFR* amplification defined as EGFR/CEP7 ratio ≥2.0, or percentage of tumor cells containing ≥15 *EGFR* signals or large clusters ≥10%.

Definitions of *HER-2* amplification [3]

(1) Low-level *HER-2* amplification defined as percentage of tumor cells containing ≥4 *HER-2* signals ≥40%;

(2) High-level *HER-2* amplification defined as HER-2/CEP17 ratio ≥2.0, or percentage of tumor cells containing ≥15 *HER-2* signals or large clusters ≥10%.

Definitions of *PDGFRA* amplification [4]

(1) Low-level *PDGFRA* amplification defined as 4≤average number of *PDGFRA* signals per tumor cell<10;

(2) High-level *PDGFRA* amplification defined as average number of *PDGFRA* signals per tumor cell≥10.

Definitions of *CCND1* amplification [5]

(1) Low-level *CCND1* amplification defined as 2≤CCND1/CEP11 ratio<4, or 4≤average number of *CCND1* signals per tumor cell<8;

(2) High-level *CCND1* amplification defined as CCND1/CEP11 ratio≥4, or average number of *CCND1* signals per tumor cell≥8.

Definitions of *SOX2* amplification [6, 7]

(1) Low-level *SOX2* amplification were defined as samples with additional 2–9 *SOX2* signals exceeding the number of CEP3 signals in at least 30% of tumor cells;

(2) High-level *SOX2* amplification were defined as samples with additional ≥10 *SOX2* signals displaying a cluster-like formation in at least 30% of tumor cells.

Definitions of *CDKN2A* deletion [8]

Cases with ≥20% of nuclei lacking both signals of the *CDKN2A* probe and showing at least one signal for the *CEP 9* probe were defined as *CDKN2A* homozygous deletion.

Definitions of *PTEN* deletion [9]

Hemizygous deletion of *PTEN* gene was defined as one red *PTEN* signal and two green *CEP10* signals in a tumor cell. Homozygous deletion of *PTEN* gene was defined as none red *PTEN* signal but with at least one green *CEP10* signal in a tumor cell . *PTEN* deletion was defined as percentage of tumor cells with hemizygous deletion or homozygous deletion of *PTEN* ≥63%.

REFERENCES

1. Schildhaus HU, Heukamp LC, Merkelbach-Bruse S, Riesner K, Schmitz K, Binot E, Paggen E, Albus K, Schulte W, Ko YD, Schlesinger A, Ansen S, Engel-Riedel W, et al. Definition of a fluorescence in-situ hybridization score identifies high- and low-level FGFR1 amplification types in squamous cell lung cancer. Mod Pathol. 2012; 25:1473-1480.
2. Tran TN, Selinger CI, Kohonen-Corish MR, McCaughan BC, Kennedy CW, O'Toole SA and Cooper WA. Fibroblast growth factor receptor 1 (FGFR1) copy number is an independent prognostic factor in non-small cell lung cancer. Lung Cancer. 2013; 81:462-467.

3. Hirsch FR, Varella-Garcia M, McCoy J, West H, Xavier AC, Gumerlock P, Bunn PA, Jr., Franklin WA, Crowley J and Gandara DR. Increased epidermal growth factor receptor gene copy number detected by fluorescence in situ hybridization associates with increased sensitivity to gefitinib in patients with bronchioloalveolar carcinoma subtypes: a Southwest Oncology Group Study. J Clin Oncol. 2005; 23:6838-6845.
4. Ramos AH, Dutt A, Mermel C, Perner S, Cho J, Lafargue CJ, Johnson LA, Stiedl AC, Tanaka KE, Bass AJ, Barretina J, Weir BA, Beroukhim R, et al. Amplification of chromosomal segment 4q12 in non-small cell lung cancer. Cancer Biol Ther. 2009; 8:2042-2050.
5. Roy PG, Pratt N, Purdie CA, Baker L, Ashfield A, Quinlan P and Thompson AM. High CCND1 amplification identifies a group of poor prognosis women with estrogen receptor positive breast cancer. Int J Cancer. 2010; 127:355-360.
6. Schrock A, Goke F, Wagner P, Bode M, Franzen A, Braun M, Huss S, Agaimy A, Ihrler S, Menon R, Kirsten R, Kristiansen G, Bootz F, et al. Sex determining region Y-box 2 (SOX2) amplification is an independent indicator of disease recurrence in sinonasal cancer. Plos One. 2013; 8:e59201.
7. Wilbertz T, Wagner P, Petersen K, Stiedl AC, Scheble VJ, Maier S, Reischl M, Mikut R, Altorki NK, Moch H, Fend F, Staebler A, Bass AJ, et al. SOX2 gene amplification and protein overexpression are associated with better outcome in squamous cell lung cancer. Mod Pathol. 2011; 24:944-953.
8. Illei PB, Rusch VW, Zakowski MF and Ladanyi M. Homozygous deletion of CDKN2A and codeletion of the methylthioadenosine phosphorylase gene in the majority of pleural mesotheliomas. Clin Cancer Res. 2003; 9:2108-2113.
9. Yanagawa N, Leduc C, Kohler D, Saieg MA, John T, Sykes J, Yoshimoto M, Pintilie M, Squire J, Shepherd FA and Tsao MS. Loss of phosphatase and tensin homolog protein expression is an independent poor prognostic marker in lung adenocarcinoma. J Thorac Oncol. 2012; 7:1513-1521.
