# Supplementary material for: Genetic alteration profiling of patients with resected squamous cell lung carcinomas
Source: Oncotarget. 2016 Apr 29;7(24):36590–601. doi: 10.18632/oncotarget.9096 (PMC5095023; doi:10.18632/oncotarget.9096)
Supplement: Supplementary file 2 [file oncotarget-07-36590-s002.docx]

**Supplementary Table 1.** Summary of mutations identified by Ion Torrent targeted sequencing in SqCLC

| **Gene** | **Mutation** | **Amino acid change** | **No.** |
| --- | --- | --- | --- |
| *TP53* | c.310C>T | p.Q104X | 1 |
|  | c.392A>T | p.N131I | 1 |
|  | c.394A>T | p.K132X | 1 |
|  | c.404G>T | p.C135F | 1 |
|  | c.464C>T | p.T155I | 1 |
|  | c.467G>C | p.R156P | 2 |
|  | c.469G>T | p.V157F | 5 |
|  | c.472C>G | p.R158G | 1 |
|  | c.473G>C | p.R158P | 1 |
|  | c.473G>T | p.R158L | 5 |
|  | c.475G>C | p.A159P | 2 |
|  | c.487T>C | p.Y163H | 1 |
|  | c.488A>G | p.Y163C | 1 |
|  | c.517G>C | p.V173L | 1 |
|  | c.518T>G | p.V173G | 1 |
|  | c.523C>G | p.R175G | 1 |
|  | c.524G>A | p.R175H | 3 |
|  | c.527G>A | p.C176Y | 1 |
|  | c.527G>T | p.C176F | 1 |
|  | c.535C>T | p.H179Y | 1 |
|  | c.536A>G | p.H179R | 3 |
|  | c.536A>T | p.H179L | 3 |
|  | c.542G>C | p.R181P | 1 |
|  | c.574C>T | p.Q192X | 1 |
|  | c.578A>G | p.H193R | 1 |
|  | c.586C>T | p.R196X | 1 |
|  | c.592G>T | p.E198X | 1 |
|  | c.610G>T | p.E204X | 1 |
|  | c.637C>T | p.R213X | 1 |
|  | c.659A>G | p.Y220C | 4 |
|  | c.707A>G | p.Y236C | 1 |
|  | c.710T>A | p.M237K | 1 |
|  | c.711G>A | p.M237I | 1 |
|  | c.711G>T | p.M237I | 1 |
|  | c.713G>T | p.C238F | 1 |
|  | c.715A>G | p.N239D | 1 |
|  | c.725G>A | p.C242Y | 1 |
|  | c.725G>T | p.C242F | 1 |
|  | c.726C>A | p.C242X | 1 |
|  | c.730G>T | p.G244C | 2 |
|  | c.731G>A | p.G244D | 1 |
|  | c.733G>T | p.G245C | 1 |
|  | c.734G>A | p.G245D | 2 |
|  | c.734G>T | p.G245V | 1 |
|  | c.742C>T | p.R248W | 1 |
|  | c.746G>T | p.R249M | 1 |
|  | c.747G>T | p.R249S | 4 |
|  | c.794T>C | p.L265P | 1 |
|  | c.796G>T | p.G266X | 2 |
|  | c.797G>A | p.G266E | 1 |
|  | c.797G>T | p.G266V | 3 |
|  | c.814G>C | p.V272L | 1 |
|  | c.820G>T | p.V274F | 1 |
|  | c.824G>A | p.C275Y | 1 |
|  | c.832C>T | p.P278S | 1 |
|  | c.841G>A | p.D281N | 1 |
|  | c.853G>A | p.E285K | 1 |
|  | c.856G>A | p.E286K | 1 |
|  | c.871A>T | p.K291X | 1 |
|  | c.880delG | p.294del_frameshift | 1 |
|  | c.880G>T | p.E294X | 1 |
|  | c.892G>T | p.E298X | 2 |
|  | c.916C>T | p.R306X | 2 |
|  | c.1006G>T | p.E336X | 1 |
|  | c.1010G>T | p.R337L | 1 |
|  | c.1024C>T | p.R342X | 1 |
| *CDKN2A* | c.181G>T | p.E61X | 2 |
|  | c.199G>A | p.G67S | 1 |
|  | c.205G>T | p.E69X | 1 |
|  | c.220G>T | p.D74Y | 1 |
|  | c.238C>T | p.R80X | 2 |
|  | c.247C>T | p.H83Y | 1 |
|  | c.250G>T | p.D84Y | 1 |
|  | c.322G>T | p.D108Y | 1 |
|  | c.358G>T | p.E120X | 3 |
|  | c.387C>G | p.Y129X | 1 |
|  | c.389T>C | p.L130P | 1 |
| *PIK3CA* | c.1624G>A | p.E542K | 6 |
|  | c.1633G>A | p.E545K | 6 |
|  | c.3140A>T | p.H1047L | 1 |
|  | c.3140A>G | p.H1047R | 1 |
| *KRAS* | c.34G>T | p.G12C | 1 |
|  | c.35G>T | p.G12D | 1 |
|  | c.35G>A | p.G12D | 5 |
|  | c.176C>A | p.A59E | 1 |
| *EGFR* | c.2235_2249del15 | p.745del_frameshift | 1 |
|  | c.2236_2250del15 | p.746_750delELREA | 1 |
|  | c.2300_2301insCAGCGTGGA | p.767ins_frameshift | 1 |
|  | c.2303G>T | p.S768I | 1 |
|  | c.2573T>G | p.L858R | 1 |
| *FBXW7* | c.832C>T | p.R278X | 1 |
|  | c.1177C>T | p.R393X | 1 |
|  | c.1394G>T | p.R465L | 1 |
|  | c.1513C>G | p.R505G | 1 |
| *PTEN* | c.314G>A | p.C105Y | 1 |
|  | c.388C>T | p.R130X | 1 |
|  | c.494G>A | p.G165E | 1 |
|  | c.963delA | p.321del_frameshift | 1 |
| *FGFR3* | c.742C>T | p.R248C | 1 |
|  | c.746C>G | p.S249C | 2 |
| *AKT1* | c.49G>A | p.E17K | 1 |
|  | c.145G>A | p.E49K | 1 |
| *KIT* | c.2447A>T | p.D816V | 1 |
