# Supplementary material for: Genetic alteration profiling of patients with resected squamous cell lung carcinomas
Source: Oncotarget. 2016 Apr 29;7(24):36590–601. doi: 10.18632/oncotarget.9096 (PMC5095023; doi:10.18632/oncotarget.9096)
Supplement: Supplementary file 3 [file oncotarget-07-36590-s003.docx]

**Supplementary Table 2**. The Association between Clinicopathologic Characteristics and Mutational Status of *EGFR*, *KRAS*, *PIK3CA*, *CDKN2A* and *TP53* in 157 SqCLC.

| Variables | N (%) | ***EGFR*** | | |  | ***KRAS*** | | |  | ***PIK3CA*** | | |  | ***CDKN2A*** | | |  | ***TP53*** | | |
| --- | --- | --- | --- | --- | --- | --- | --- | --- | --- | --- | --- | --- | --- | --- | --- | --- | --- | --- | --- | --- |
|  |  | Mut | Wild | *P* |  | Mut | Wild | *P* |  | Mut | Wild | *P* |  | Mut | Wild | *P* |  | Mut | Wild | *P* |
| Total |  | 5 | 152 |  |  | 7 | 150 |  |  | 14 | 143 |  |  | 14 | 143 |  |  | 88 | 69 |  |
| **Age (years)** |  |  |  | 0.928 |  |  |  | 0.545 |  |  |  | 1.000 |  |  |  | 0.220 |  |  |  | 0.305 |
| < 65 | 107 (68.2) | 4 | 103 |  |  | 6 | 101 |  |  | 10 | 97 |  |  | 7 | 100 |  |  | 57 | 50 |  |
| ≥ 65 | 50 (31.8) | 1 | 49 |  |  | 1 | 49 |  |  | 4 | 46 |  |  | 7 | 43 |  |  | 31 | 19 |  |
| **Sex** |  |  |  | **0.000** |  |  |  | 1.000 |  |  |  | 0.650 |  |  |  | 1.000 |  |  |  | **0.024** |
| Male | 145 (92.4) | 1 | 144 |  |  | 6 | 139 |  |  | 12 | 133 |  |  | 13 | 132 |  |  | 85 | 60 |  |
| Female | 12 (7.6) | 4 | 8 |  |  | 1 | 11 |  |  | 2 | 10 |  |  | 1 | 11 |  |  | 3 | 9 |  |
| **Smoking status**^a^ |  |  |  | **0.000** |  |  |  | 1.000 |  |  |  | 1.000 |  |  |  | 1.000 |  |  |  | **0.019** |
| Never smoker | 17 (10.8) | 4 | 13 |  |  | 1 | 16 |  |  | 2 | 15 |  |  | 2 | 15 |  |  | 5 | 12 |  |
| Former smoker | 25 (15.9) | 0 | 25 |  |  | 1 | 24 |  |  | 3 | 22 |  |  | 4 | 21 |  |  | 12 | 13 |  |
| Current smoker | 115 (73.2) | 1 | 114 |  |  | 5 | 110 |  |  | 9 | 106 |  |  | 8 | 107 |  |  | 71 | 44 |  |
| **Histology**^b^ |  |  |  | 1.000 |  |  |  | 1.000 |  |  |  | 0.959 |  |  |  | 0.959 |  |  |  | 0.340 |
| Squamous | 151 (96.2) | 5 | 146 |  |  | 7 | 144 |  |  | 14 | 137 |  |  | 14 | 137 |  |  | 83 | 68 |  |
| Adenosquamous | 1 (0.6) | 0 | 1 |  |  | 0 | 1 |  |  | 0 | 1 |  |  | 0 | 1 |  |  | 1 | 0 |  |
| Squamous with small cell | 2 (1.3) | 0 | 2 |  |  | 0 | 2 |  |  | 0 | 2 |  |  | 0 | 2 |  |  | 2 | 0 |  |
| Squamous with basaloid | 3 (1.9) | 0 | 3 |  |  | 0 | 3 |  |  | 0 | 3 |  |  | 0 | 3 |  |  | 2 | 1 |  |
| **Differentiation**^c^ |  |  |  | 0.919 |  |  |  | 0.371 |  |  |  | **0.041** |  |  |  | 0.861 |  |  |  | 0.990 |
| Well | 9 (5.7) | 1 | 8 |  |  | 0 | 9 |  |  | 2 | 7 |  |  | 0 | 9 |  |  | 3 | 6 |  |
| Moderate | 74 (46.5) | 1 | 72 |  |  | 2 | 71 |  |  | 9 | 64 |  |  | 7 | 66 |  |  | 43 | 30 |  |
| Poor | 75 (47.8) | 3 | 72 |  |  | 5 | 70 |  |  | 3 | 72 |  |  | 7 | 68 |  |  | 42 | 33 |  |
| **pT stage** |  |  |  |  |  |  |  | 0.354 |  |  |  | 0.696 |  |  |  | 0.478 |  |  |  | 0.088 |
| T1 | 17 (10.2) | 2 | 14 | 0.150 |  | 0 | 16 |  |  | 2 | 14 |  |  | 0 | 16 |  |  | 5 | 11 |  |
| T2 | 98 (62.4) | 2 | 96 |  |  | 6 | 92 |  |  | 8 | 90 |  |  | 9 | 89 |  |  | 61 | 37 |  |
| T3 | 32 (19.7) | 1 | 30 |  |  | 0 | 31 |  |  | 2 | 29 |  |  | 3 | 28 |  |  | 17 | 14 |  |
| T4 | 12 (7.6) | 0 | 12 |  |  | 1 | 11 |  |  | 2 | 10 |  |  | 2 | 10 |  |  | 5 | 7 |  |
| **pN stage**^d^ |  |  |  | 0.373 |  |  |  | 0.849 |  |  |  | 0.783 |  |  |  | 0.403 |  |  |  | 0.537 |
| N0 | 73 (46.5) | 1 | 72 |  |  | 4 | 69 |  |  | 7 | 66 |  |  | 8 | 65 |  |  | 39 | 31 |  |
| N1 | 41 (26.1) | 1 | 40 |  |  | 2 | 39 |  |  | 3 | 38 |  |  | 1 | 40 |  |  | 22 | 19 |  |
| N2 | 43 (27.4) | 3 | 40 |  |  | 1 | 42 |  |  | 4 | 39 |  |  | 5 | 38 |  |  | 27 | 18 |  |
| **pTNM stage**^e^ |  |  |  | 0.539 |  |  |  | 0.529 |  |  |  | 0.631 |  |  |  | 0.849 |  |  |  | 0.870 |
| I | 53 (33.8) | 1 | 52 |  |  | 4 | 49 |  |  | 6 | 47 |  |  | 6 | 47 |  |  | 29 | 24 |  |
| II | 46 (29.3) | 1 | 45 |  |  | 2 | 44 |  |  | 2 | 44 |  |  | 2 | 44 |  |  | 26 | 20 |  |
| III | 57 (36.3) | 3 | 54 |  |  | 1 | 56 |  |  | 6 | 51 |  |  | 6 | 51 |  |  | 32 | 25 |  |
| IV | 1 (0.7) | 0 | 1 |  |  | 0 | 1 |  |  | 0 | 1 |  |  | 0 | 1 |  |  | 1 | 0 |  |

a, Never smoker *vs.* Former smoker and Current somker; b, squamous *vs.* the others; c, Well and Moderate *vs.* Poor; d, N0 *vs*. N1 and N2; e, I and II *vs.* III and IV. Abbreviations: mut, mutation.
