# Supplementary material for: Genetic alteration profiling of patients with resected squamous cell lung carcinomas
Source: Oncotarget. 2016 Apr 29;7(24):36590–601. doi: 10.18632/oncotarget.9096 (PMC5095023; doi:10.18632/oncotarget.9096)
Supplement: Supplementary file 4 [file oncotarget-07-36590-s004.docx]

**Supplementary Table** **3.** Univariate and multivariate analyses of prognostic factors for DFS and OS.

| **Variable** | **Category** | **Univariate** | | | | | | |  | **Multivariate** | | | | | | |
| --- | --- | --- | --- | --- | --- | --- | --- | --- | --- | --- | --- | --- | --- | --- | --- | --- |
|  |  | **DFS** | | |  | **OS** | | |  | **DFS** | | |  | **OS** | | |
|  |  | **HR** | **95%CI** | ***P*** |  | **HR** | **95%CI** | ***P*** |  | **HR** | **95%CI** | ***P*** |  | **HR** | **95%CI** | ***P*** |
| Age | ≥65 *vs.* <65 (ref) | 1.776 | 1.121-2.815 | **0.015** |  | 2.216 | 1.278-3.844 | **0.005** |  | 1.764 | 1.109-2.806 | **0.016** |  | 2.177 | 1.253-3.782 | **0.006** |
| Differentiation | well+moderate *vs.* poor (ref) | 1.747 | 1.105-2.763 | **0.017** |  | 1.825 | 1.040-3.204 | **0.036** |  | 1.534 | 0.955-2.465 | 0.077 |  | 1.566 | 0.875-2.805 | 0.131 |
| TNM stage | III+IV *vs.* I+II (ref) | 2.435 | 1.544-3.840 | **0.000** |  | 2.493 | 1.434-4.334 | **0.001** |  | 2.150 | 1.339-3.452 | **0.002** |  | 2.200 | 1.240-3.905 | **0.007** |
| *TP53* mut | mut *vs.* wt (ref) | 1.404 | 0.082-2.236 | 0.153 |  | 1.490 | 0.844-2.630 | 0.169 |  | - | - | - |  | - | - | - |
| *CDKN2A* mut | mut *vs.* wt (ref) | 0.838 | 0.364-1.931 | 0.678 |  | 0.843 | 0.304-2.341 | 0.734 |  | - | - | - |  | - | - | - |
| *PIK3CA* mut | mut *vs.* wt (ref) | 1.315 | 0.631-2.737 | 0.465 |  | 1.447 | 0.617-3.393 | 0.395 |  | - | - | - |  | - | - | - |
| *KRAS* mut | mut *vs.* wt (ref) | 0.560 | 0.137-2.283 | 0.418 |  | 0.913 | 0.222-3.758 | 0.899 |  | - | - | - |  | - | - | - |
| *EGFR* mut | mut *vs.* wt (ref) | 0.296 | 0.041-2.134 | 0.227 |  | 0.047 | 0.000-22.976 | 0.333 |  | - | - | - |  | - | - | - |
| *FGFR1* amp | amp+ *vs.* amp- (ref) | 1.041 | 0.561-1.931 | 0.898 |  | 1.026 | 0.482-2.185 | 0.946 |  | - | - | - |  | - | - | - |
| *EGFR* amp | amp+ *vs.* amp- (ref) | 1.341 | 0.737-2.438 | 0.337 |  | 1.181 | 0.555-2.513 | 0.666 |  | - | - | - |  | - | - | - |
| *HER2* amp | amp+ *vs.* amp- (ref) | 0.776 | 0.337-1.787 | 0.551 |  | 0.529 | 0.165-1.699 | 0.285 |  | - | - | - |  | - | - | - |
| *PDGFRA* amp | amp+ *vs.* amp- (ref) | 0.576 | 0.210-1.579 | 0.284 |  | 0.727 | 0.226-2.333 | 0.592 |  | - | - | - |  | - | - | - |
| *CCND1* amp | amp+ *vs.* amp- (ref) | 1.339 | 0.722-2.483 | 0.354 |  | 1.225 | 0.576-2.607 | 0.598 |  | - | - | - |  | - | - | - |
| *SOX2* amp | amp+ *vs.* amp- (ref) | 1.332 | 0.827-2.144 | 0.238 |  | 0.940 | 0.514-1.716 | 0.839 |  | - | - | - |  | - | - | - |
| *CDKN2A* del | del *vs.* normal (ref) | 1.437 | 0.853-2.418 | 0.173 |  | 1.185 | 0.620-2.263 | 0.608 |  | - | - | - |  | - | - | - |
| *PTEN* del | del *vs.* normal (ref) | 0.839 | 0.442-1.590 | 0.590 |  | 0.511 | 0.203-1.288 | 0.155 |  | - | - | - |  | - | - | - |
| PTEN loss | loss *vs.* normal (ref) | 0.935 | 0.592-1.475 | 0.772 |  | 0.966 | 0.555-1.682 | 0.904 |  | - | - | - |  | - | - | - |
| PD-L1 postive | positive *vs.* negative (ref) | 0.547 | 0.341-0.878 | **0.012** |  | 0.626 | 0.355-1.104 | 0.106 |  | 0.610 | 0.377-0.987 | **0.044** |  | 0.719 | 0.401-1.287 | 0.266 |
| VEGFR2 positive | positive *vs.* negative (ref) | 1.579 | 0.832-2.993 | 0.162 |  | 1.136 | 0.553-2.333 | 0.729 |  | - | - | - |  | - | - | - |

Abbreviations: mut, mutation; wt, wild-type; amp, amplification; del, deletion; DFS, disease-free survival; OS, overall survival; HR, hazard ratio; CI, confidence interval; ref, reference.
