# Supplementary material for: Genetic alteration profiling of patients with resected squamous cell lung carcinomas
Source: Oncotarget. 2016 Apr 29;7(24):36590–601. doi: 10.18632/oncotarget.9096 (PMC5095023; doi:10.18632/oncotarget.9096)
Supplement: Supplementary file 5 [file oncotarget-07-36590-s005.docx]

**Supplementary Table 4**. The Association between Clinicopathologic Characteristics and Copy Number Alterations in 157 SqCLC.

|  | N (%) | ***FGFR1*** | | |  | ***EGFR*** | | |  | ***HER2*** | | |  | ***PDGFRA*** | | |
| --- | --- | --- | --- | --- | --- | --- | --- | --- | --- | --- | --- | --- | --- | --- | --- | --- |
|  |  | Amp | Disomy | *P* |  | Amp | Disomy | *P* |  | Amp | Disomy | *P* |  | Amp | Disomy | *P* |
| Total |  | 25 | 132 |  |  | 22 | 135 |  |  | 15 | 142 |  |  | 12 | 145 |  |
| **Age (years)** |  |  |  | 0.358 |  |  |  | 0.322 |  |  |  | 0.872 |  |  |  | 1.000 |
| < 65 | 107 (68.2) | 19 | 88 |  |  | 17 | 90 |  |  | 11 | 96 |  |  | 8 | 99 |  |
| ≥ 65 | 50 (31.8) | 6 | 44 |  |  | 5 | 45 |  |  | 4 | 46 |  |  | 4 | 46 |  |
| **Sex** |  |  |  | 0.247 |  |  |  | 1.000 |  |  |  | 1.000 |  |  |  | 0.637 |
| Male | 145 (92.4) | 25 | 120 |  |  | 20 | 125 |  |  | 14 | 131 |  |  | 12 | 133 |  |
| Female | 12 (7.6) | 0 | 12 |  |  | 2 | 10 |  |  | 1 | 11 |  |  | 0 | 12 |  |
| **Smoking status**^a^ |  |  |  | 0.121 |  |  |  | 1.000 |  |  |  | 0.444 |  |  |  | 0.440 |
| Never smoker | 17 (10.8) | 0 | 17 |  |  | 2 | 15 |  |  | 3 | 14 |  |  | 0 | 17 |  |
| Former smoker | 25 (15.9) | 3 | 22 |  |  | 3 | 22 |  |  | 1 | 24 |  |  | 2 | 23 |  |
| Current smoker | 115 (73.2) | 22 | 93 |  |  | 17 | 98 |  |  | 11 | 104 |  |  | 10 | 105 |  |
| **Histology**^b^ |  |  |  | 1.000 |  |  |  | 1.000 |  |  |  | 0.917 |  |  |  | 0.915 |
| Squamous | 151 (96.2) | 24 | 127 |  |  | 21 | 130 |  |  | 15 | 136 |  |  | 12 | 139 |  |
| Adenosquamous | 1 (0.6) | 0 | 1 |  |  | 1 | 0 |  |  | 0 | 1 |  |  | 0 | 1 |  |
| Squamous with small cell | 2 (1.3) | 0 | 2 |  |  | 0 | 2 |  |  | 0 | 2 |  |  | 0 | 2 |  |
| Squamous with basaloid | 3 (1.9) | 0 | 2 |  |  | 0 | 3 |  |  | 0 | 3 |  |  | 0 | 3 |  |
| **Differentiation**^c^ |  |  |  | 0.644 |  |  |  | 0.248 |  |  |  | 0.928 |  |  |  | 0.872 |
| Well | 9 (5.7) | 13 | 8 |  |  | 1 | 8 |  |  | 1 | 8 |  |  | 1 | 8 |  |
| Moderate | 74 (46.5) | 11 | 62 |  |  | 13 | 60 |  |  | 7 | 66 |  |  | 5 | 68 |  |
| Poor | 75 (47.8) | 1 | 62 |  |  | 8 | 67 |  |  | 7 | 68 |  |  | 6 | 69 |  |
| **pT stage** |  |  |  | 0.816 |  |  |  | 0.454 |  |  |  | 0.299 |  |  |  | 0.740 |
| T1 | 17 (10.2) | 2 | 14 |  |  | 1 | 15 |  |  | 0 | 16 |  |  | 1 | 15 |  |
| T2 | 98 (62.4) | 16 | 82 |  |  | 15 | 83 |  |  | 11 | 87 |  |  | 8 | 90 |  |
| T3 | 32 (19.7) | 6 | 25 |  |  | 3 | 28 |  |  | 4 | 27 |  |  | 3 | 28 |  |
| T4 | 12 (7.6) | 1 | 11 |  |  | 3 | 9 |  |  | 0 | 12 |  |  | 0 | 12 |  |
| **pN stage**^d^ |  |  |  | 0.056 |  |  |  | 0.916 |  |  |  | 0.577 |  |  |  | 0.800 |
| N0 | 73 (46.5) | 16 | 57 |  |  | 10 | 63 |  |  | 8 | 65 |  |  | 6 | 67 |  |
| N1 | 41 (26.1) | 3 | 38 |  |  | 5 | 36 |  |  | 3 | 38 |  |  | 1 | 40 |  |
| N2 | 43 (27.4) | 6 | 37 |  |  | 7 | 36 |  |  | 4 | 39 |  |  | 5 | 38 |  |
| **pTNM stage**^e^ |  |  |  | 0.312 |  |  |  | 0.372 |  |  |  | 0.761 |  |  |  | 0.967 |
| I | 53 (33.8) | 12 | 41 |  |  | 8 | 45 |  |  | 5 | 48 |  |  | 5 | 48 |  |
| II | 46 (29.3) | 6 | 40 |  |  | 4 | 42 |  |  | 5 | 41 |  |  | 2 | 44 |  |
| III | 57 (36.3) | 7 | 50 |  |  | 10 | 47 |  |  | 5 | 52 |  |  | 5 | 52 |  |
| IV | 1 (0.7) | 0 | 1 |  |  | 0 | 1 |  |  | 0 | 1 |  |  | 0 | 1 |  |

a, Never smoker *vs.* Former smoker and Current somker; b, squamous *vs.* the others; c, Well and Moderate *vs.* Poor; d, N0 *vs.* N1 and N2; e, I and II *vs.* III and IV. Abbreviations: Amp, amplification.

**Supplementary Table 4**. The Association between Clinicopathologic Characteristics and Copy Number Alterations in 157 SqCLC (Continued).

|  | N (%) | ***CCND1*** | | |  | ***SOX2*** | | |  | ***CDKN2A*** | | |  | ***PTEN*** | | |
| --- | --- | --- | --- | --- | --- | --- | --- | --- | --- | --- | --- | --- | --- | --- | --- | --- |
|  |  | Amp | Disomy | *P* |  | Amp | Disomy | *P* |  | Del | Disomy | *P* |  | Del | Disomy | *P* |
| Total |  | 22 | 135 |  |  | 49 | 108 |  |  | 34 | 123 |  |  | 26 | 131 |  |
| **Age (years)** |  |  |  | 0.322 |  |  |  | 0.606 |  |  |  | 0.447 |  |  |  | 0.293 |
| < 65 | 107 (68.2) | 17 | 90 |  |  | 32 | 75 |  |  | 25 | 82 |  |  | 20 | 87 |  |
| ≥ 65 | 50 (31.8) | 5 | 45 |  |  | 17 | 33 |  |  | 9 | 41 |  |  | 6 | 44 |  |
| **Sex** |  |  |  | 0.307 |  |  |  | 0.146 |  |  |  | 1.000 |  |  |  | 0.694 |
| Male | 145 (92.4) | 22 | 123 |  |  | 48 | 97 |  |  | 31 | 114 |  |  | 25 | 120 |  |
| Female | 12 (7.6) | 0 | 12 |  |  | 1 | 11 |  |  | 3 | 9 |  |  | 1 | 11 |  |
| **Smoking status**^a^ |  |  |  | 0.514 |  |  |  | 0.067 |  |  |  | 0.610 |  |  |  | 0.636 |
| Never smoker | 17 (10.8) | 1 | 16 |  |  | 2 | 15 |  |  | 5 | 12 |  |  | 4 | 13 |  |
| Former smoker | 25 (15.9) | 3 | 22 |  |  | 8 | 17 |  |  | 7 | 18 |  |  | 4 | 21 |  |
| Current smoker | 115 (73.2) | 18 | 97 |  |  | 39 | 76 |  |  | 22 | 93 |  |  | 18 | 97 |  |
| **Histology**^b^ |  |  |  | 0.429 |  |  |  | 0.218 |  |  |  | **0.026** |  |  |  | 1.000 |
| Squamous | 151 (96.2) | 20 | 131 |  |  | 49 | 102 |  |  | 30 | 121 |  |  | 25 | 126 |  |
| Adenosquamous | 1 (0.6) | 0 | 1 |  |  | 0 | 1 |  |  | 1 | 0 |  |  | 0 | 1 |  |
| Squamous with small cell | 2 (1.3) | 1 | 1 |  |  | 0 | 2 |  |  | 0 | 2 |  |  | 0 | 2 |  |
| Squamous with basaloid | 3 (1.9) | 1 | 2 |  |  | 0 | 3 |  |  | 3 | 0 |  |  | 1 | 2 |  |
| **Differentiation**^c^ |  |  |  | 0.252 |  |  |  | 0.888 |  |  |  | 0.495 |  |  |  | 0.542 |
| Well | 9 (5.7) | 13 | 62 |  |  | 0 | 9 |  |  | 1 | 8 |  |  | 11 | 64 |  |
| Moderate | 74 (46.5) | 8 | 65 |  |  | 26 | 47 |  |  | 15 | 58 |  |  | 14 | 59 |  |
| Poor | 75 (47.8) | 1 | 8 |  |  | 23 | 52 |  |  | 18 | 57 |  |  | 1 | 8 |  |
| **pT stage** |  |  |  | 0.658 |  |  |  | 0.385 |  |  |  | 0.767 |  |  |  | 0.594 |
| T1 | 17 (10.2) | 2 | 13 |  |  | 8 | 8 |  |  | 3 | 13 |  |  | 1 | 15 |  |
| T2 | 98 (62.4) | 12 | 86 |  |  | 28 | 70 |  |  | 21 | 77 |  |  | 17 | 81 |  |
| T3 | 32 (19.7) | 6 | 25 |  |  | 9 | 22 |  |  | 6 | 25 |  |  | 5 | 26 |  |
| T4 | 12 (7.6) | 1 | 11 |  |  | 4 | 8 |  |  | 4 | 8 |  |  | 3 | 9 |  |
| **pN stage**^d^ |  |  |  | 0.137 |  |  |  | 0.674 |  |  |  | 0.644 |  |  |  | 0.969 |
| N0 | 73 (46.5) | 7 | 66 |  |  | 24 | 49 |  |  | 17 | 56 |  |  | 12 | 61 |  |
| N1 | 41 (26.1) | 9 | 32 |  |  | 11 | 30 |  |  | 10 | 31 |  |  | 5 | 36 |  |
| N2 | 43 (27.4) | 6 | 37 |  |  | 14 | 29 |  |  | 7 | 36 |  |  | 9 | 34 |  |
| **pTNM stage**^e^ |  |  |  | 0.171 |  |  |  | 0.971 |  |  |  | 0.860 |  |  |  | 0.287 |
| I | 53 (33.8) | 5 | 48 |  |  | 21 | 32 |  |  | 14 | 39 |  |  | 8 | 45 |  |
| II | 46 (29.3) | 6 | 40 |  |  | 10 | 36 |  |  | 7 | 39 |  |  | 6 | 40 |  |
| III | 57 (36.3) | 11 | 46 |  |  | 17 | 40 |  |  | 13 | 44 |  |  | 12 | 45 |  |
| IV | 1 (0.7) | 0 | 1 |  |  | 1 | 0 |  |  | 0 | 1 |  |  | 0 | 1 |  |

a, Never smoker *vs.* Former smoker and Current somker; b, squamous *vs.* the others; c, Well and Moderate *vs.* Poor; d, N0 *vs.* N1 and N2; e, I and II *vs.* III and IV. Abbreviations: Amp, amplification; Del, deletion.
