# Supplementary material for: Genetic alteration profiling of patients with resected squamous cell lung carcinomas
Source: Oncotarget. 2016 Apr 29;7(24):36590–601. doi: 10.18632/oncotarget.9096 (PMC5095023; doi:10.18632/oncotarget.9096)
Supplement: Supplementary file 6 [file oncotarget-07-36590-s006.docx]

**Supplementary Table 5**. The Association between Clinicopathologic Characteristics and Expression of PTEN, PD-L1 and VEGFR2 in 157 SqCLC.

|  | N (%) | **PTEN** | | |  | **PD-L1** | | |  | **VEGFR2** | | |
| --- | --- | --- | --- | --- | --- | --- | --- | --- | --- | --- | --- | --- |
|  |  | Losted | Absent | *P* |  | + | - | *P* |  | + | - | *P* |
| Total |  | 67 | 90 |  |  | 74 | 83 |  |  | 126 | 31 |  |
| **Age (years)** |  |  |  | **0.050** |  |  |  | 0.221 |  |  |  | 0.707 |
| < 65 | 107 (68.2) | 40 | 67 |  |  | 54 | 53 |  |  | 85 | 22 |  |
| ≥ 65 | 50 (31.8) | 27 | 23 |  |  | 20 | 50 |  |  | 41 | 9 |  |
| **Sex** |  |  |  | 0.058 |  |  |  | 0.836 |  |  |  | 0.512 |
| Male | 145 (92.4) | 65 | 80 |  |  | 68 | 77 |  |  | 115 | 30 |  |
| Female | 12 (7.6) | 2 | 10 |  |  | 6 | 6 |  |  | 11 | 1 |  |
| **Smoking status**^a^ |  |  |  | 0.699 |  |  |  | 0.300 |  |  |  | 0.580 |
| Never smoker | 17 (10.8) | 8 | 9 |  |  | 6 | 11 |  |  | 15 | 2 |  |
| Former smoker | 25 (15.9) | 10 | 15 |  |  | 13 | 12 |  |  | 21 | 4 |  |
| Current smoker | 115 (73.2) | 49 | 66 |  |  | 55 | 60 |  |  | 90 | 25 |  |
| **Histology**^b^ |  |  |  | 0.372 |  |  |  | 0.268 |  |  |  | 0.169 |
| Squamous | 151 (96.2) | 66 | 85 |  |  | 73 | 78 |  |  | 123 | 28 |  |
| Adenosquamous | 1 (0.6) | 0 | 1 |  |  | 0 | 1 |  |  | 1 | 0 |  |
| Squamous with small cell | 2 (1.3) | 0 | 2 |  |  | 0 | 1 |  |  | 2 | 0 |  |
| Squamous with basaloid | 3 (1.9) | 1 | 4 |  |  | 0 | 2 |  |  | 0 | 3 |  |
| **Differentiation**^c^ |  |  |  | 0.745 |  |  |  | 0.396 |  |  |  | 0.093 |
| Well | 9 (5.7) | 5 | 4 |  |  | 38 | 37 |  |  | 56 | 19 |  |
| Moderate | 74 (46.5) | 31 | 42 |  |  | 34 | 39 |  |  | 61 | 12 |  |
| Poor | 75 (47.8) | 31 | 44 |  |  | 2 | 7 |  |  | 9 | 0 |  |
| **pT stage** |  |  |  | 0.220 |  |  |  | 0.080 |  |  |  | 0.868 |
| T1 | 17 (10.2) | 4 | 12 |  |  | 6 | 10 |  |  | 13 | 3 |  |
| T2 | 98 (62.4) | 45 | 53 |  |  | 54 | 44 |  |  | 80 | 18 |  |
| T3 | 32 (19.7) | 15 | 16 |  |  | 10 | 21 |  |  | 25 | 6 |  |
| T4 | 12 (7.6) | 3 | 9 |  |  | 4 | 8 |  |  | 8 | 4 |  |
| **pN stage**^d^ |  |  |  | 0.059 |  |  |  | 0.250 |  |  |  | 0.638 |
| N0 | 73 (46.5) | 37 | 36 |  |  | 38 | 35 |  |  | 59 | 14 |  |
| N1 | 41 (26.1) | 15 | 26 |  |  | 21 | 20 |  |  | 31 | 10 |  |
| N2 | 43 (27.4) | 15 | 28 |  |  | 15 | 28 |  |  | 36 | 7 |  |
| **pTNM stage**^e^ |  |  |  | 0.054 |  |  |  | **0.015** |  |  |  | 0.820 |
| I | 53 (33.8) | 26 | 27 |  |  | 28 | 25 |  |  | 42 | 11 |  |
| II | 46 (29.3) | 22 | 24 |  |  | 26 | 20 |  |  | 38 | 8 |  |
| III | 57 (36.3) | 19 | 38 |  |  | 19 | 38 |  |  | 45 | 12 |  |
| IV | 1 (0.7) | 0 | 1 |  |  | 1 | 0 |  |  | 1 | 0 |  |

a, Never smoker *vs.* Former smoker and Current somker; b, squamous *vs.* the others; c, Well and Moderate *vs.* Poor; d, N0 *vs.* N1 and N2; e, I and II *vs.* III and IV.
