# Supplementary material for: Genetic alteration profiling of patients with resected squamous cell lung carcinomas
Source: Oncotarget. 2016 Apr 29;7(24):36590–601. doi: 10.18632/oncotarget.9096 (PMC5095023; doi:10.18632/oncotarget.9096)
Supplement: Supplementary file 7 [file oncotarget-07-36590-s007.docx]

**Supplementary Table 6.** Correlation between the various main molecular alterations

|  |  | Mut | | | | | | | | | |  | Amp/del | | | | | | | |  | Expression | | |
| --- | --- | --- | --- | --- | --- | --- | --- | --- | --- | --- | --- | --- | --- | --- | --- | --- | --- | --- | --- | --- | --- | --- | --- | --- |
|  |  | *TP53* | *CDKN2A* | *PIK3CA* | *KRAS* | *EGFR* | *FBXW7* | *PTEN* | *FGFR3* | *AKT1* | *KIT* |  | *FGFR1* | *EGFR* | *HER2* | *PDGFRA* | *CCND1* | *SOX2* | *CDKN2A* | *PTEN* |  | *PTEN* | *PD-L1* | *VEGFR2* |
| Mut | *TP53* |  |  |  |  |  |  |  |  |  |  |  |  |  |  |  |  |  |  |  |  |  |  |  |
|  | *CDKN2A* | 0.583 |  |  |  |  |  |  |  |  |  |  |  |  |  |  |  |  |  |  |  |  |  |  |
|  | *PIK3CA* | 0.399 | 1.000 |  |  |  |  |  |  |  |  |  |  |  |  |  |  |  |  |  |  |  |  |  |
|  | *KRAS* | **0.044** | 1.000 | 1.000 |  |  |  |  |  |  |  |  |  |  |  |  |  |  |  |  |  |  |  |  |
|  | *EGFR* | 0.170 | 1.000 | 1.000 | 1.000 |  |  |  |  |  |  |  |  |  |  |  |  |  |  |  |  |  |  |  |
|  | *FBXW7* | 1.000 | 0.314 | 1.000 | 0.168 | 1.000 |  |  |  |  |  |  |  |  |  |  |  |  |  |  |  |  |  |  |
|  | *PTEN* | 1.000 | 1.000 | 1.000 | 1.000 | 1.000 | 1.000 |  |  |  |  |  |  |  |  |  |  |  |  |  |  |  |  |  |
|  | *FGFR3* | 0.504 | 1.000 | 1.000 | 1.000 | 1.000 | 1.000 | 1.000 |  |  |  |  |  |  |  |  |  |  |  |  |  |  |  |  |
|  | *AKT1* | 0.192 | 1.000 | 1.000 | 1.000 | 1.000 | 1.000 | 1.000 | 1.000 |  |  |  |  |  |  |  |  |  |  |  |  |  |  |  |
|  | *KIT* | 0.439 | 1.000 | 1.000 | 1.000 | 1.000 | 1.000 | 1.000 | 1.000 | 1.000 |  |  |  |  |  |  |  |  |  |  |  |  |  |  |
|  |  |  |  |  |  |  |  |  |  |  |  |  |  |  |  |  |  |  |  |  |  |  |  |  |
| Amp/del | *FGFR1* | 0.084 | 0.469 | 0.700 | 0.309 | 1.000 | 1.000 | 1.000 | 1.000 | 1.000 | 1.000 |  |  |  |  |  |  |  |  |  |  |  |  |  |
|  | *EGFR* | 0.645 | 0.418 | 1.000 | 0.594 | 0.144 | 0.457 | 0.457 | 1.000 | 1.000 | 1.000 |  | 0.352 |  |  |  |  |  |  |  |  |  |  |  |
|  | *HER2* | 1.000 | 0.364 | 0.364 | 0.512 | 1.000 | 1.000 | 1.000 | 1.000 | 1.000 | 1.000 |  | 0.263 | 0.696 |  |  |  |  |  |  |  |  |  |  |
|  | *PDGFRA* | 0.552 | 0.077 | 0.291 | 1.000 | 1.000 | 1.000 | 1.000 | 1.000 | 1.000 | 1.000 |  | 0.408 | 0.377 | 0.092 |  |  |  |  |  |  |  |  |  |
|  | *CCND1* | 1.000 | 0.221 | 1.000 | 0.594 | 1.000 | 1.000 | 1.000 | 1.000 | 0.261 | 1.000 |  | 1.000 | 1.000 | 0.444 | 0.067 |  |  |  |  |  |  |  |  |
|  | *SOX2* | 0.608 | 0.765 | 1.000 | 1.000 | 0.326 | 1.000 | 1.000 | 1.000 | 1.000 | 1.000 |  | 1.000 | 0.806 | 0.558 | 0.518 | 0.806 |  |  |  |  |  |  |  |
|  | *CDKN2A* | 0.846 | 0.305 | 0.505 | 1.000 | 1.000 | 1.000 | 0.578 | 1.000 | 1.000 | 1.000 |  | 0.793 | 0.263 | 0.194 | 1.000 | 0.786 | 1.000 |  |  |  |  |  |  |
|  | *PTEN* | 0.388 | 0.705 | 0.468 | 0.090 | 0.591 | 0.519 | 0.519 | 0.305 | 1.000 | 1.000 |  | 0.769 | 1.000 | 1.000 | 0.219 | 1.000 | 0.817 | 1.000 |  |  |  |  |  |
|  |  |  |  |  |  |  |  |  |  |  |  |  |  |  |  |  |  |  |  |  |  |  |  |  |
| Expression | *PTEN* | 0.516 | 0.098 | 0.397 | 0.137 | 1.000 | 0.313 | **0.031** | 0.508 | 0.508 | 1.000 |  | **0.027** | 0.819 | **0.014** | 0.763 | **0.003** | 0.490 | **0.011** | 0.128 |  |  |  |  |
|  | *PD-L1* | 0.152 | 1.000 | 0.786 | 0.256 | 1.000 | 1.000 | 0.623 | 0.498 | 1.000 | 0.471 |  | 0.665 | 0.647 | 0.787 | **0.013** | 0.647 | **0.024** | 0.252 | 0.670 |  | 1.000 |  |  |
|  | *VEGFR2* | 0.550 | **0.008** | 0.305 | 0.346 | 0.584 | 0.586 | 0.586 | 0.357 | 0.357 | 1.000 |  | 0.586 | 1.000 | 0.306 | 0.462 | **0.045** | 0.387 | 0.330 | 0.417 |  | 0.106 | 0.322 |  |

The left below part of the matrix gives the corresponding *P* value estimate from a Fisher exact test.

Abbreviation: Mut, mutation; Amp/del, amplification/deletion.
